# A locally deployed large language model for pathology-informed and nurse-reviewed communication support in bladder cancer immunotherapy

**DOI:** 10.3389/fmed.2026.1903690

**Published:** 2026-08-10

**Authors:** Suqing Diao, Jun Zhao, Xuzhong Liu

**Affiliations:** Department of Urology, The Affiliated Huaian No. 1 People's Hospital of Nanjing Medical University, Huaian, China

**Keywords:** bladder cancer, human-in-the-loop, immune-related adverse events, immunotherapy, large language model, nursing communication, pathology-informed communication, symptom triage

## Abstract

**Background:**

Immunotherapy plays an important role in bladder cancer care, requiring ongoing patient education, symptom monitoring, and communication of pathology- and biomarker-related information. Locally deployed large language models (LLMs) may support these nurse-led activities, but their safety and clinical usability remain uncertain.

**Method:**

We conducted a human-in-the-loop evaluation of a locally deployed Qwen2-7B-Instruct model using structured Mandarin-language benchmarks. Five task categories were assessed: general immunotherapy education, pathology-informed communication, biomarker and precision pathology communication, symptom triage, and pathology-risk-informed triage. Outputs were reviewed by specialist nurses, with physician or pathologist adjudication for high-risk, discordant, or pathology-related cases. Evaluation dimensions included accuracy, completeness, readability, actionability, safety, pathology-feature recognition, uncertainty communication, red-flag recognition, escalation appropriateness, and revision burden.

**Results:**

The locally deployed model Qwen2-7B achieved an overall score of 3.83 ± 0.23 for general patient education, performing best in disease understanding and self-management but less well in warning-sign education. In pathology-informed communication, the model achieved an overall score of 3.57 ± 0.22, with stronger performance in patient-friendly translation and structured pathology-feature recognition than in risk communication and follow-up guidance. Biomarker communication represented a more challenging task (overall score 3.26 ± 0.25), with frequent needs for uncertainty statements, clinician referral, and correction of biomarker overinterpretation. In symptom triage, agreement with expert triage levels was 75.9%, red-flag recognition was 71.1%, and escalation sensitivity was 77.1%; under-triage and unsafe reassurance remained observable, particularly in high-risk irAE scenarios. Incorporating pathology-derived risk context improved triage agreement from 52.5 to 70.0% and reduced under-triage, although safety-critical errors persisted. Across all tasks, only 3.7% of outputs required no revision, whereas 31.4% required major revision or withholding. The highest review burden occurred in biomarker communication and high-risk irAE scenarios.

**Conclusions:**

A locally deployed Qwen2-7B-based LLM can support nurse-reviewed communication in bladder cancer immunotherapy, particularly for standardized education and explanation of structured pathology information. However, biomarker communication, pathology-derived risk interpretation, and immune-related symptom escalation remain safety-sensitive tasks requiring structured nurse oversight and clinician backup. These findings support locally deployed LLMs as human-in-the-loop nursing support tools rather than autonomous communication or triage systems within precision uro-oncology care.

## 1 Introduction

Bladder cancer is one of the most common urological malignancies worldwide, with more than 600,000 new cases estimated in 2022 and a long-term disease course characterized by recurrence, surveillance, and stage-dependent treatment complexity (1). Immunotherapy has become an essential component of bladder cancer care across the disease continuum. Intravesical bacillus Calmette–Guérin (BCG) remains a cornerstone for intermediate- and high-risk non–muscle-invasive bladder cancer, whereas immune checkpoint inhibitors have reshaped the management of advanced or metastatic urothelial carcinoma and are increasingly being evaluated in perioperative and biomarker-guided settings (2–5). However, the clinical value of immunotherapy depends not only on treatment selection, but also on whether patients understand treatment goals, report warning symptoms early, and receive timely escalation when immune-related or treatment-related toxicities occur.

This implementation challenge is particularly relevant to oncology nursing. Nurses are central to patient education, symptom monitoring, telephone or outpatient follow-up, and early recognition of immune-related adverse events (irAEs). Current guidelines emphasize that irAEs may affect multiple organ systems and require early assessment, appropriate grading, treatment interruption when needed, and escalation for severe symptoms (6, 7). In practice, patients may describe symptoms vaguely, underestimate severity, or fail to connect delayed toxicities with immunotherapy. Nurses therefore serve as the first safety filter, translating complex oncology information into understandable instructions while identifying red flags such as dyspnea, severe diarrhea, jaundice, high fever, altered mental status, persistent bleeding, or rapidly worsening general condition (8).

At the same time, bladder cancer care is increasingly shaped by pathology-derived risk factors and molecular information. Tumor stage, grade, lymph node status, lymphovascular invasion, carcinoma *in situ*, surgical margin status, and variant histology influence prognosis, follow-up intensity, and treatment discussion (9, 10). In parallel, immune and molecular biomarkers such as PD-L1 expression, MSI/MMR status, tumor mutational burden, FGFR alteration, circulating tumor DNA, and tumor immune microenvironment features are being investigated or used to support patient stratification and therapeutic planning (11–13). These developments create new communication needs: patients must be informed accurately, but without deterministic claims, unsupported treatment recommendations, or overinterpretation of uncertain biomarkers.

Large language models (LLMs) have shown potential for patient education, oncology question answering, documentation, and clinical communication support, but their outputs may be incomplete, overconfident, insufficiently contextualized, or unsafe in high-stakes scenarios (14–16). Recent work has emphasized the need for structured human evaluation, domain-specific review, and safety-oriented governance before LLMs are integrated into healthcare workflows (17). Locally deployed LLMs may offer additional advantages for institutional data control and privacy-sensitive clinical environments, but local deployment does not itself guarantee clinical safety (18, 19).

Despite growing interest in clinical LLMs, evidence remains limited regarding their use in bladder cancer immunotherapy nursing workflows, especially for pathology-informed communication, biomarker explanation, symptom triage, and pathology-risk-informed escalation. We therefore conducted a human-in-the-loop evaluation of a locally deployed Qwen2-7B-Instruct model as a nurse-reviewed communication support tool for bladder cancer immunotherapy care. The study assessed whether the model could generate useful patient-facing drafts, where human revision was required, and how nurse review contributed to safety governance across education, precision pathology communication, and immune-related symptom triage. Rather than proposing a new general evaluation framework, this single-center study evaluates the practical utility and safety limitations of a locally deployed LLM within a predefined nurse-reviewed bladder cancer immunotherapy communication workflow.

## 2 Materials and methods

### 2.1 Study design

This study was designed as a human-in-the-loop evaluation of a locally deployed large language model (LLM) for nurse-reviewed communication support in bladder cancer immunotherapy care. The model was evaluated as a draft-generation and communication-assistance tool, not as an autonomous diagnostic, pathology interpretation, biomarker interpretation, or triage system. The evaluation included five task categories: general patient education, pathology-informed communication, biomarker and precision pathology communication, symptom triage, and pathology-risk-informed triage. All tasks were developed in Mandarin Chinese to reflect patient-facing communication in the intended clinical workflow. The overall process included standardized prompt-based generation, independent nurse review, clinician or pathologist adjudication for high-risk or discordant outputs, and final classification of revision burden. Detailed procedures are provided in Supplementary Methods 1–7.

### 2.2 Model deployment and comparator models

The primary model was Qwen2-7B-Instruct, deployed locally in a controlled computing environment. The local deployment was intended to support hospital-controlled inference and reduce the need to transmit clinical communication content to external services. The model generated draft responses only, and no output was considered suitable for patient-facing use without human review. To contextualize the local model's performance, comparator LLMs were evaluated using identical task inputs and prompt templates. The comparator models included Qwen2.5-72B-Instruct, DeepSeek-V3.1, Llama-4-Maverick, Gemini 3.1 Pro, Claude Opus 4.8, and GPT-5.5. Model access mode, version information, inference date, and generation settings are described in Supplementary Methods 2, 3.

### 2.3 Evaluation datasets

Five structured Mandarin-language evaluation datasets were developed to reflect common communication tasks encountered in bladder cancer immunotherapy care. Benchmark items were constructed from routine nursing education topics, typical follow-up questions, structured postoperative pathology concepts, biomarker communication needs, and symptom scenarios relevant to intravesical bacillus Calmette–Guérin (BCG) or immune checkpoint inhibitor therapy. Before evaluation, the datasets were reviewed for clinical plausibility, patient-facing relevance, and diversity of clinical risk levels. The baseline characteristics of the source patients used for evaluation scenario development are summarized in Table 1.

**Table 1:** Baseline characteristics of source patients used for evaluation scenario development.

Characteristic	Overall *N* = 120	NMIBC/BCG *N* = 45	Postoperative MIBC *N* = 35	Advanced/ICI *N* = 40
Age, median (IQR), years	69 (65–74)	70 (67–73)	70 (62–74)	69 (65–73)
Age ≥75 years	27 (22.5%)	10 (22.2%)	9 (25.7%)	8 (20.0%)
Male sex	82 (68.3%)	30 (66.7%)	26 (74.3%)	26 (65.0%)
ECOG 0–1	98 (81.7%)	39 (86.7%)	29 (82.9%)	30 (75.0%)
Former/current smoker	89 (74.2%)	32 (71.1%)	27 (77.1%)	30 (75.0%)
High-grade disease	116 (96.7%)	42 (93.3%)	35 (100.0%)	39 (97.5%)
Variant histology	28 (23.3%)	8 (17.8%)	9 (25.7%)	11 (27.5%)
CIS	31 (25.8%)	19 (42.2%)	7 (20.0%)	5 (12.5%)
LVI	49 (40.8%)	5 (11.1%)	21 (60.0%)	23 (57.5%)
Positive lymph node status	31 (25.8%)	0 (0.0%)	9 (25.7%)	22 (55.0%)
Metastatic disease	21 (17.5%)	0 (0.0%)	0 (0.0%)	21 (52.5%)
PD-L1 tested	71 (59.2%)	6 (13.3%)	25 (71.4%)	40 (100.0%)
FGFR alteration detected	20 (16.7%)	2 (4.4%)	8 (22.9%)	10 (25.0%)
ctDNA/MRD assessed	36 (30.0%)	3 (6.7%)	13 (37.1%)	20 (50.0%)

The patient education benchmark included 56 questions covering disease understanding, immunotherapy purpose, treatment process, common adverse effects, warning signs, and home self-management. The pathology-informed communication set included 42 structured postoperative pathology scenarios involving histological type, tumor grade, pathological stage, muscle invasion, carcinoma *in situ*, lymphovascular invasion, surgical margin status, lymph node status, multifocality, and treatment-context integration. The biomarker and precision pathology communication set included 36 scenarios related to PD-L1 expression, MSI/MMR status, tumor mutational burden, FGFR alteration, ctDNA/MRD, and the tumor immune microenvironment.

The symptom triage set comprised 54 case vignettes representing low-risk routine symptoms, moderate-risk symptoms, high-risk immune-related adverse event scenarios, postoperative or post-treatment complications, and ambiguous scenarios requiring clinical-context interpretation. A separate pathology-risk-informed triage set included 40 paired scenarios in which symptom-only prompts were compared with otherwise identical prompts containing pathology-derived risk information. This paired design was used to assess whether risk context altered escalation recommendations. Detailed dataset construction procedures and representative examples are provided in Supplementary Methods 4.

### 2.4 Prompting and output generation

All models received standardized task-specific prompts designed to generate nurse-reviewable patient-facing communication drafts rather than autonomous clinical advice. Each prompt included a clinical boundary statement specifying that the model should not independently make diagnostic, pathology-interpretation, biomarker-interpretation, treatment-selection, or emergency-triage decisions.

For patient education, pathology, and biomarker tasks, outputs were instructed to use clear patient-friendly language, avoid unsupported prognostic or treatment claims, communicate uncertainty where appropriate, and encourage discussion with the treating clinical team. Pathology and biomarker prompts further required the model to avoid deterministic statements regarding recurrence risk, treatment response, or individual prognosis. For triage tasks, outputs were required to identify relevant red flags, provide safety-netting instructions, and recommend one of four nursing-oriented triage levels: self-care or routine monitoring; contact with the nurse team within 24–48 h; urgent clinic or hospital assessment; or immediate emergency escalation.

Each model generated one response per item under predefined inference settings. Complete prompt templates, model settings, and technical generation procedures are provided in Supplementary Methods 3.

### 2.5 Human review and adjudication

Each model output was independently reviewed by two specialist nurses selected from a panel of three nurses with experience in urological oncology or oncology nursing. Reviewers assessed task-specific dimensions, including clinical correctness, completeness, patient-facing clarity, actionability, safety, and, where applicable, red-flag recognition and appropriateness of escalation advice. Reviewers also recorded predefined safety concerns and independently assigned a revision category.

Outputs were classified as no edit, minor edit, major edit, or withhold/rewrite. No edit indicated that the output could be used after review without modification. Minor edit indicated that the core clinical content was acceptable but wording, tone, clarity, or formatting required revision. Major edit indicated that clinically important content, risk communication, uncertainty statements, safety-netting, red-flag warnings, or escalation advice required correction. Withhold/rewrite indicated that the output was unsafe, misleading, outside the intended communication-support scope, or unsuitable for patient-facing use after routine editing. Agreement between nurse reviewers did not necessarily indicate that an output required no revision; reviewers could agree that minor or major revision was required.

Clinician or pathologist adjudication was triggered by substantial reviewer disagreement and by prespecified high-risk conditions, including high-risk immune-related adverse event scenarios, possible severe under-triage, complex pathology findings, biomarker overinterpretation, unsupported treatment recommendations, or other treatment-boundary concerns. A urologist, medical oncologist, or pathologist was selected for adjudication according to the clinical content of the item. Detailed reviewer instructions, adjudication criteria, and revision definitions are provided in Supplementary Methods 5.

### 2.6 Evaluation metrics and outcomes

General education outputs were scored for accuracy, completeness, readability, actionability, empathy, and safety. Pathology-informed communication was assessed for key pathology feature recognition, accuracy of explanation, patient-friendly translation, risk communication appropriateness, treatment-boundary awareness, and follow-up actionability. Biomarker communication was assessed for concept accuracy, patient-friendly translation, uncertainty communication, treatment-boundary awareness, clinician referral/actionability, and safety. Symptom triage and pathology-risk-informed triage were assessed for triage agreement, red-flag recognition, escalation sensitivity, under-triage, severe under-triage, unsafe reassurance, and safety-netting adequacy. The main workflow outcome was human revision burden. Full scoring definitions are provided in Supplementary Methods 6.

### 2.7 Statistical analysis and ethics

Continuous ratings were summarized as mean ± standard deviation, and categorical variables were reported as counts and percentages. Analyses were primarily descriptive because the study was designed as an exploratory clinical AI evaluation. Agreement between model triage recommendations and expert reference standards was calculated as the proportion of exact matches. Under-triage was defined as a recommendation below the expert reference level, and severe under-triage was defined as failure to recommend urgent or emergency assessment for high-risk scenarios. Inter-rater reliability was assessed using Cohen's kappa, weighted kappa, or intraclass correlation coefficient, as appropriate. The study used de-identified or structured clinical scenarios. No model output was directly delivered to patients without human review. Additional statistical and governance details are provided in Supplementary Methods 7.

## 3 Results

### 3.1 Overview of evaluation datasets and human-in-the-loop workflow

**Figure 1:**
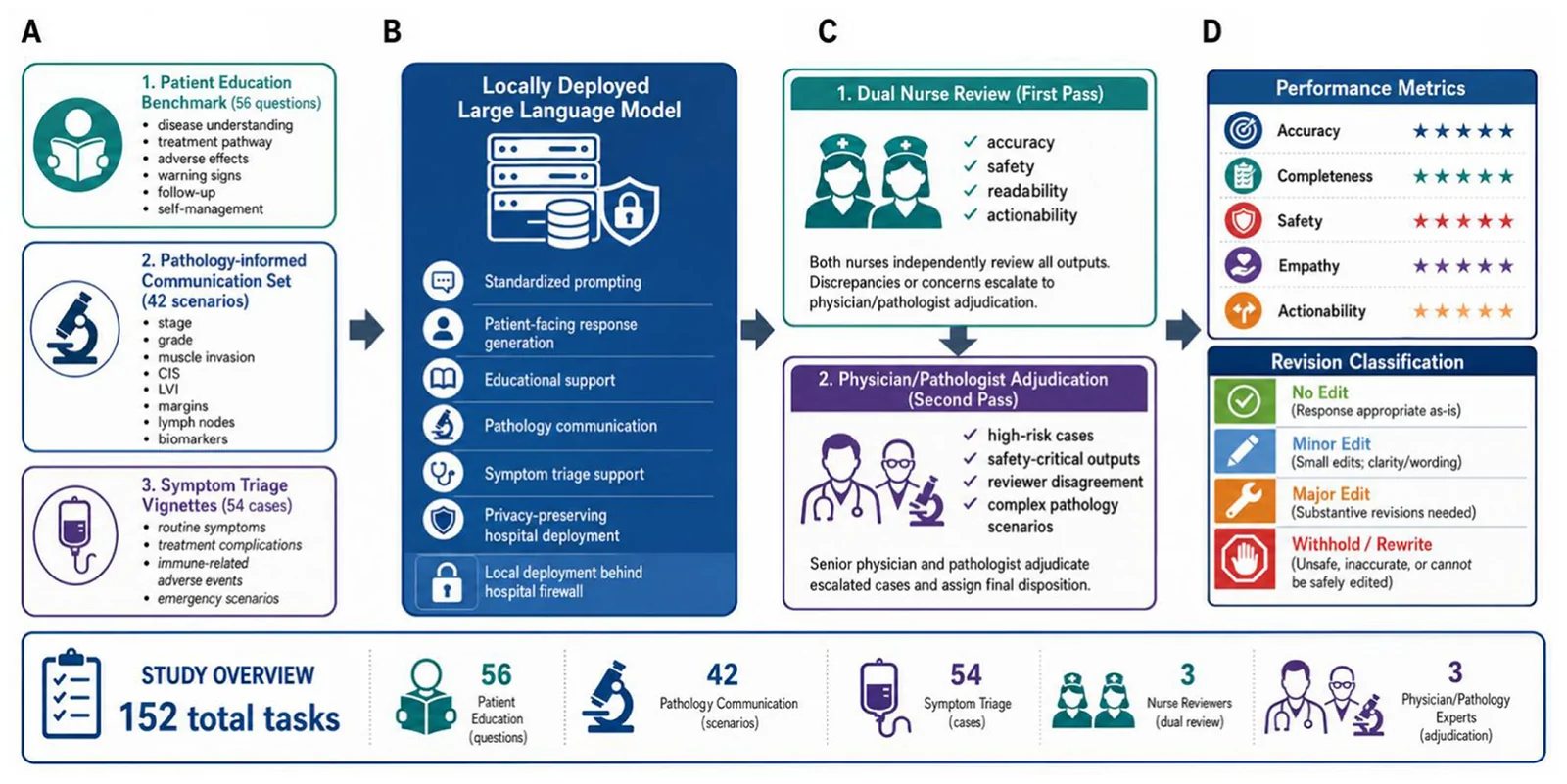
Study design and human-in-the-loop evaluation workflow for LLM-supported bladder cancer immunotherapy care. The core benchmark comprised 152 Mandarin-language items: 56 patient education questions, 42 pathology-informed communication scenarios, and 54 symptom triage vignettes. An additional 36 biomarker and precision-pathology communication scenarios were included in the cross-task revision-burden analysis, yielding 188 non-overlapping model outputs. A separate paired analysis included 40 pathology-risk-informed triage scenarios evaluated using symptom-only and pathology-risk-informed prompts; these scenarios were excluded from the 152-item core benchmark and the 188-output revision-burden analysis. Outputs underwent independent review by two specialist nurses, with clinician or pathologist adjudication for discordant, high-risk, or safety-sensitive cases. **(A)** Evaluation datasets. **(B)** Locally deployed large language model. **(C)** Human-in-the-loop review. **(D)** Final scoring and revision classification.

In this evaluation framework, the final benchmark comprised 152 items distributed across three task families: 56 patient education questions, 42 pathology-informed communication scenarios, and 54 symptom triage vignettes (Figure 1). The patient education benchmark covered seven domains, including disease understanding, treatment pathways, expected adverse effects, immune-related warning signs, follow-up and surveillance, home self-management, and emotional support. These items were stratified as 18 basic, 26 intermediate, and 12 safety-critical questions.

The pathology-informed communication set included 42 structured postoperative pathology summaries or pathology-derived scenarios. These scenarios were designed to evaluate whether the locally deployed LLM could translate clinically relevant pathology information into nurse-reviewed patient-facing explanations without exceeding the scope of communication support. The set included scenarios involving tumor type and grade, pathological stage and muscle invasion, carcinoma *in situ*, multifocality, lymphovascular invasion, margin status, lymph node status, and biomarker or precision-pathology information. Of these, 6 were classified as basic, 22 as intermediate, and 14 as safety-critical.

The symptom triage vignette set comprised 54 structured cases spanning routine post-treatment urinary symptoms, potential treatment-related complications, possible immune-related adverse events, high-risk escalation scenarios, and ambiguous scenarios requiring consideration of pathology-risk context. Reference triage endpoints were prespecified using a four-level framework: self-care/routine monitoring, contact with the nurse team within 24–48 h, urgent clinic or hospital assessment, and immediate emergency escalation. The triage set included 7 low-risk, 19 moderate-risk, and 28 high-risk vignettes.

All LLM outputs were generated using standardized prompts and were evaluated under a human-in-the-loop workflow. Each output was independently reviewed by two nurse reviewers selected from a panel of three urology/oncology nurses. Initial nurse-level agreement was observed in 120 of 152 outputs, corresponding to 78.9%. Physician or pathology adjudication was required for 75 outputs, corresponding to 49.3%, including all safety-critical or high-risk items, outputs with nurse-review disagreement, and complex pathology or biomarker communication scenarios. Adjudication was performed by a urologic oncologist, medical oncologist, or urologic pathologist according to the clinical content of the item.

For workflow evaluation, each output was assigned to one of four revision categories: no edit, minor edit, major edit, or withhold/rewrite. The classification was used to quantify the level of human oversight required before any patient-facing use. In the dataset, the revision categories were retained as item-level raw variables to support later analyses of human revision burden and safety-critical error patterns.

### 3.2 Performance on general bladder cancer immunotherapy education

**Figure 2:**
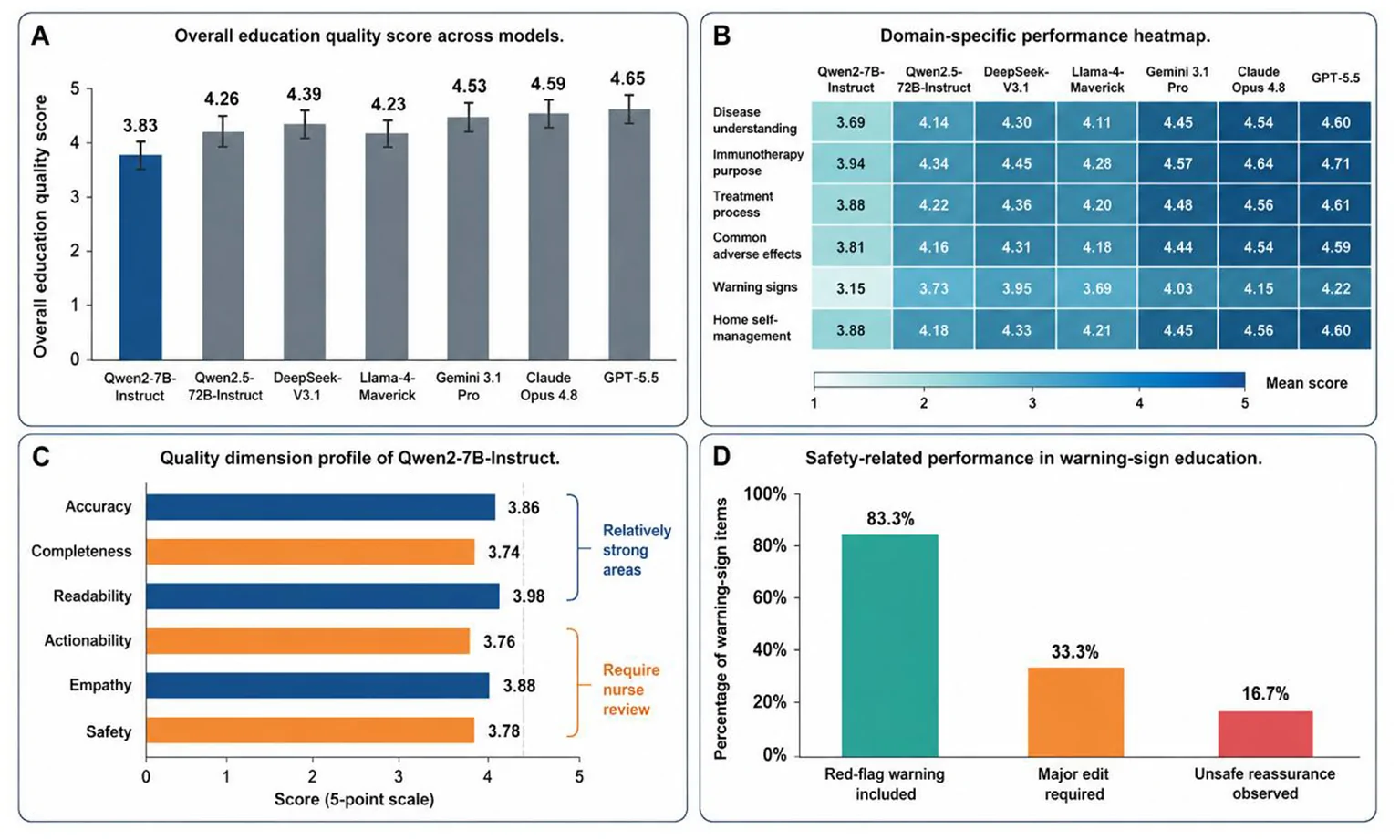
Performance of LLMs on general bladder cancer immunotherapy patient education. **(A)** Overall education quality scores across the locally deployed Qwen2-7B-Instruct model and six comparator LLMs. **(B)** Domain-specific performance heatmap across six patient education domains, including disease understanding, immunotherapy purpose, treatment process, common adverse effects, warning signs, and home self-management. **(C)** Quality dimension profile of the locally deployed Qwen2-7B-Instruct model, including accuracy, completeness, readability, actionability, empathy, and safety. **(D)** Safety-related performance of Qwen2-7B-Instruct in warning-sign education, including red-flag warning inclusion, major revision requirement, and unsafe reassurance.

The patient education benchmark included 56 bladder cancer immunotherapy-related questions across six domains: disease understanding, immunotherapy purpose, treatment process, common adverse effects, warning signs, and home self-management. The locally deployed Qwen2-7B-Instruct model achieved a mean overall education quality score of 3.83 ± 0.23 on a 5-point scale. In comparison, higher overall scores were observed for the larger or proprietary comparator models, including Qwen2.5-72B-Instruct, DeepSeek-V3.1, Llama-4-Maverick, Gemini 3.1 Pro, Claude Opus 4.8, and GPT-5.5, with mean overall scores ranging from 4.23 ± 0.16 to 4.65 ± 0.14 (Figure 2A; Table 2).

**Table 2:** Performance of LLMs on the patient education benchmark.

Model	Overall score, mean ±SD	Accuracy	Completeness	Readability	Actionability	Empathy	Safety
Qwen2-7B-Instruct	3.83 ± 0.23	3.86	3.74	3.98	3.76	3.88	3.78
Qwen2.5-72B-Instruct	4.26 ± 0.14	4.28	4.27	4.32	4.23	4.29	4.2
DeepSeek-V3.1	4.39 ± 0.13	4.44	4.38	4.5	4.33	4.42	4.37
Llama-4-Maverick	4.23 ± 0.16	4.27	4.23	4.32	4.17	4.24	4.22
Gemini 3.1 Pro	4.53 ± 0.14	4.52	4.52	4.64	4.46	4.56	4.46
Claude Opus 4.8	4.59 ± 0.14	4.6	4.51	4.71	4.54	4.67	4.51
GPT-5.5	4.65 ± 0.14	4.68	4.58	4.76	4.63	4.69	4.63

Scores were rated on a 5-point scale across six dimensions: accuracy, completeness, readability, actionability, empathy, and safety. Overall score was calculated as the mean of the six dimension scores.

Across education domains, Qwen2-7B-Instruct performed best in standardized and low-risk educational topics, including disease understanding, immunotherapy purpose, and home self-management. Its domain-level scores were highest for disease understanding (4.01 ± 0.06), immunotherapy purpose (3.98 ± 0.06), and home self-management (3.95 ± 0.03). By contrast, the lowest performance was observed in the warning signs domain (3.42 ± 0.05), where responses more frequently required clarification of red-flag symptoms and escalation thresholds (Figure 2B).

Analysis of quality dimensions showed that Qwen2-7B-Instruct generated relatively readable and patient-friendly responses. Among the six evaluated dimensions, readability was the highest-scoring dimension (3.98), followed by empathy (3.88) and accuracy (3.86). Lower scores were observed for completeness (3.74), actionability (3.76), and safety (3.78), suggesting that the local model was generally capable of producing understandable educational content but required nurse review when outputs involved comprehensive adverse-event counseling or specific next-step instructions (Figure 2C; Table 2).

Safety-related analysis of the warning-sign education subset further highlighted the need for human oversight. For Qwen2-7B-Instruct, red-flag warning information was included in 83.3% of warning-sign items. However, 33.3% of warning-sign outputs required major revision, and unsafe reassurance was observed in 16.7% of these items (Figure 2D). These findings indicate that while the locally deployed model may support nurse-reviewed patient education for routine bladder cancer immunotherapy topics, escalation-related content should not be released without structured clinical review.

Taken together, these findings indicate that the model had relatively greater utility for standardized, non-urgent educational content, such as disease understanding, treatment purpose, and home self-management. However, this pattern did not extend to warning-sign communication, in which major revision and unsafe reassurance remained observable. Therefore, favorable performance in routine education should not be extrapolated to escalation-related communication, which remained dependent on structured clinical review.

### 3.3 Pathology-informed patient education and communication performance

The pathology-informed communication set included 42 structured postoperative pathology scenarios designed to assess whether the locally deployed Qwen2-7B-Instruct model could translate pathology-derived information into nurse-reviewed, patient-facing explanations. The scenarios covered histological type, tumor grade, pathological stage, muscle invasion, carcinoma *in situ*, lymphovascular invasion, surgical margin status, lymph node status, multifocality, and treatment-context integration.

**Figure 3:**
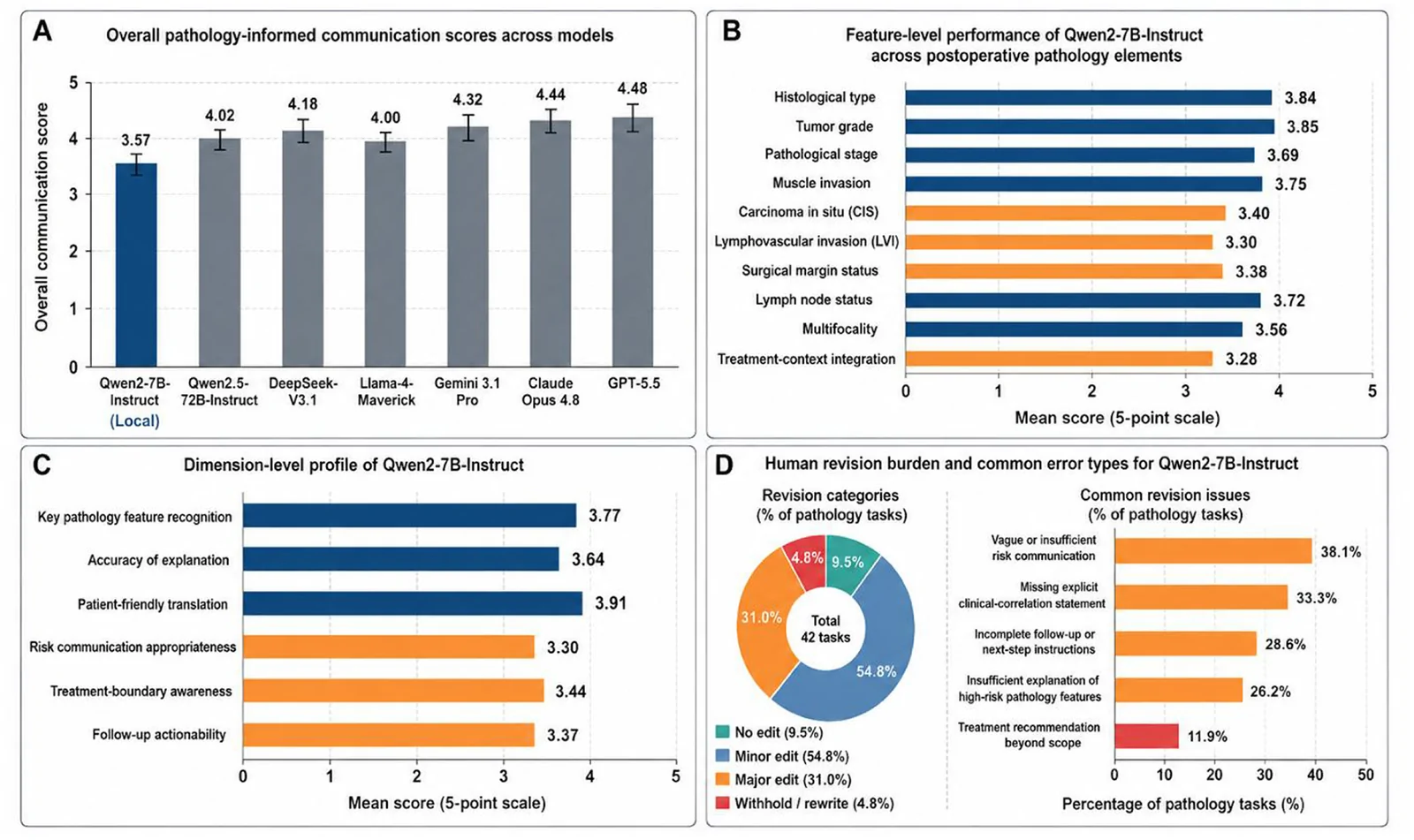
Pathology-informed patient education and communication performance. **(A)** Overall pathology-informed communication scores across the locally deployed Qwen2-7B-Instruct model and six comparator LLMs. **(B)** Feature-level performance of Qwen2-7B-Instruct across postoperative pathology elements, including histological type, tumor grade, pathological stage, muscle invasion, carcinoma *in situ*, lymphovascular invasion, surgical margin status, lymph node status, multifocality, and treatment-context integration. **(C)** Dimension-level profile of Qwen2-7B-Instruct across key pathology feature recognition, accuracy of explanation, patient-friendly translation, risk communication appropriateness, treatment-boundary awareness, and follow-up actionability. **(D)** Human revision burden and common error types for Qwen2-7B-Instruct in pathology-informed communication tasks.

Across all pathology-informed scenarios, Qwen2-7B-Instruct achieved a mean overall communication score of 3.57 ± 0.22 on a 5-point scale. Comparator models showed higher overall scores, ranging from 4.00 ± 0.24 for Llama-4-Maverick to 4.48 ± 0.22 for GPT-5.5 (Figure 3A; Table 3). Despite lower overall performance than larger or proprietary models, the locally deployed model demonstrated moderate utility for structured pathology communication under nurse review.

**Table 3:** Performance of LLMs on the pathology-informed communication set.

Model	Overall score, mean ±SD	Key feature recognition	Accuracy	Patient-friendly translation	Risk communication	Boundary awareness	Follow-up actionability	Major edit/rewrite (%)
Qwen2-7B-Instruct	3.57 ± 0.22	3.77	3.64	3.91	3.3	3.44	3.37	35.80
Qwen2.5-72B-Instruct	4.02 ± 0.23	4.09	4.07	4.21	3.85	4.03	3.89	23.80
DeepSeek-V3.1	4.18 ± 0.22	4.27	4.23	4.4	4.08	4.09	4.05	16.70
Llama-4-Maverick	4.00 ± 0.24	4.11	4.02	4.18	3.88	3.94	3.88	23.80
Gemini 3.1 Pro	4.32 ± 0.23	4.44	4.35	4.5	4.15	4.27	4.22	9.50
Claude Opus 4.8	4.44 ± 0.24	4.5	4.4	4.72	4.28	4.39	4.37	7.10
GPT-5.5	4.48 ± 0.22	4.57	4.46	4.62	4.36	4.45	4.45	4.80

For Qwen2-7B-Instruct, performance was strongest for patient-friendly translation score, 3.91, followed by key pathology feature recognition, 3.77, and accuracy of explanation, 3.64. Lower scores were observed for risk communication appropriateness, 3.30, treatment-boundary awareness, 3.44, and follow-up actionability, 3.37. This pattern suggests that the model was more capable of translating structured pathology terms into understandable language than of communicating risk, uncertainty, and appropriate next-step guidance (Figure 3C).

Feature-level analysis showed that Qwen2-7B-Instruct performed best for relatively structured pathology elements, including tumor grade, 3.85, histological type, 3.84, muscle invasion, 3.75, lymph node status, 3.72, and pathological stage, 3.69. By contrast, lower performance was observed for more complex or risk-sensitive features, including carcinoma *in situ*, 3.40, lymphovascular invasion, 3.30, surgical margin status, 3.38, and treatment-context integration, 3.28 (Figure 3B).

Key pathology features were recognized in 83.3% of Qwen2-7B-Instruct outputs overall. However, recognition and explanation were less consistent in complex pathology-risk communication tasks, especially those requiring integration of pathological findings with recurrence risk, immunotherapy context, or follow-up planning. This was particularly evident for treatment-context integration, where outputs more frequently required clinician correction or expansion.

Human review outcomes further supported the need for a structured oversight workflow. For Qwen2-7B-Instruct, 4 of 42 outputs, 9.5%, required no edit; 23 outputs, 54.8%, required minor edits; 13 outputs, 31.0%, required major edits; and 2 outputs, 4.8%, were classified as withhold/rewrite before patient-facing use (Figure 3D). The most frequent revision issues were vague or insufficient risk communication, 16/42, 38.1%; missing explicit clinical-correlation statements, 14/42, 33.3%; incomplete follow-up or next-step instructions, 12/42, 28.6%; and insufficient explanation of high-risk pathology features, 11/42, 26.2%. Treatment recommendations beyond the intended communication-support scope were observed in 5 outputs, 11.9%.

These findings indicate that pathology-informed communication should be interpreted as a heterogeneous task category. The model was relatively more suitable for nurse-reviewed translation of discrete and structured pathology terms, particularly tumor grade, pathological stage, muscle invasion, and lymph node status. In contrast, pathology features requiring risk interpretation or integration with recurrence risk, treatment context, or follow-up planning—including carcinoma *in situ*, lymphovascular invasion, margin status, and multifocality—remained dependent on physician or pathologist oversight. Thus, performance in structured terminology translation should not be interpreted as evidence of reliable autonomous pathology-risk communication.

### 3.4 Communication of immunotherapy-related biomarkers and precision pathology information

The biomarker and precision pathology communication set included 36 structured scenarios spanning six information types: PD-L1 expression, MSI/MMR status, tumor mutational burden, FGFR alteration, ctDNA/MRD, and tumor immune microenvironment. The purpose of this evaluation was not to assess autonomous biomarker interpretation or treatment selection, but to determine whether LLMs could communicate biomarker-derived information accurately, cautiously, and safely under nurse/clinician review.

**Figure 4:**
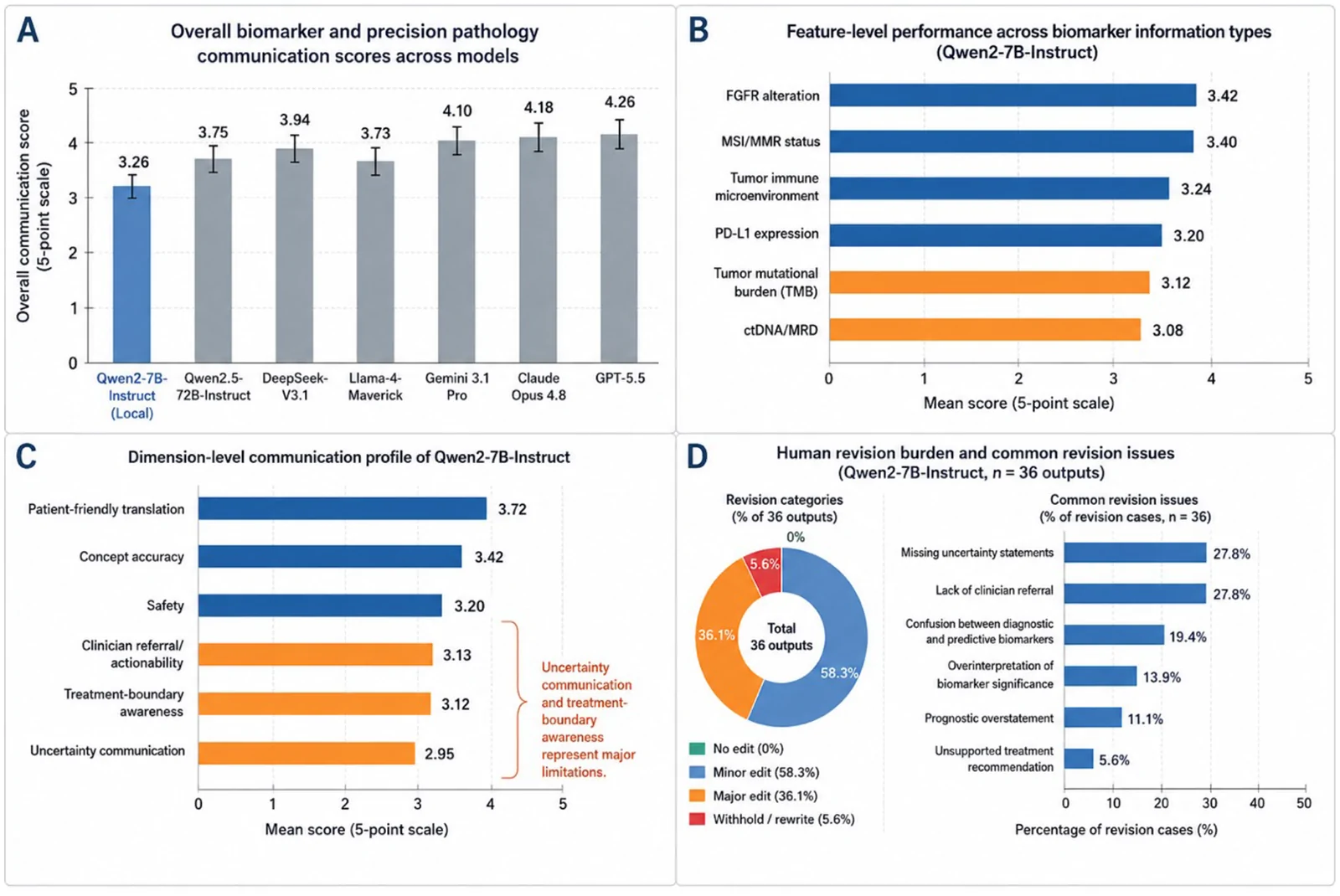
Communication of immunotherapy-related biomarkers and precision pathology information. **(A)** Overall biomarker and precision pathology communication scores across the locally deployed Qwen2-7B-Instruct model and six comparator LLMs. **(B)** Biomarker-type performance of Qwen2-7B-Instruct across PD-L1 expression, MSI/MMR status, tumor mutational burden, FGFR alteration, ctDNA/MRD, and tumor immune microenvironment scenarios. **(C)** Dimension-level profile of Qwen2-7B-Instruct across concept accuracy, patient-friendly translation, uncertainty communication, treatment-boundary awareness, clinician referral/actionability, and safety. **(D)** Human revision categories and common safety-related error types for Qwen2-7B-Instruct in biomarker and precision pathology communication tasks.

Across all biomarker communication scenarios, the locally deployed Qwen2-7B-Instruct model achieved a mean overall score of 3.26 ± 0.25 on a 5-point scale. Comparator models achieved higher mean scores, ranging from 3.73 ± 0.25 for Llama-4-Maverick to 4.26 ± 0.25 for GPT-5.5 (Figure 4A; Table 4).

**Table 4:** Performance of LLMs on the biomarker and precision pathology communication set.

Model	Overall score, mean ±SD	Concept accuracy	Patient-friendly translation	Uncertainty communication	Treatment-boundary awareness	Clinician referral/actionability	Safety	Major edit/rewrite (%)
Qwen2-7B-Instruct	3.26 ± 0.25	3.42	3.72	2.95	3.12	3.13	3.2	41.70
Qwen2.5-72B-Instruct	3.75 ± 0.24	3.87	3.94	3.59	3.76	3.66	3.67	16.70
DeepSeek-V3.1	3.94 ± 0.24	4.05	4.09	3.83	3.91	3.86	3.87	5.60
Llama-4-Maverick	3.73 ± 0.25	3.85	3.93	3.56	3.7	3.65	3.67	5.60
Gemini 3.1 Pro	4.10 ± 0.25	4.21	4.25	3.95	4.11	4.07	3.99	2.80
Claude Opus 4.8	4.18 ± 0.24	4.21	4.47	4.05	4.17	4.11	4.09	2.80
GPT-5.5	4.26 ± 0.25	4.32	4.4	4.16	4.28	4.2	4.21	2.80

For Qwen2-7B-Instruct, the highest dimension-level score was observed for patient-friendly translation (3.72), followed by concept accuracy (3.42). Lower scores were observed for safety (3.20), clinician referral/actionability (3.13), treatment-boundary awareness (3.12), and uncertainty communication (2.95). This indicates that the local model was more capable of simplifying biomarker terminology than of consistently communicating uncertainty, clinical boundaries, and the need for clinician discussion (Figure 4C). This distinction is important because biomarker-guided communication in urothelial cancer often requires nuanced explanation rather than deterministic treatment claims.

By information type, Qwen2-7B-Instruct performed best for FGFR alteration (3.42), MSI/MMR status (3.40), and tumor immune microenvironment (3.24). Lower scores were observed for PD-L1 expression (3.20), tumor mutational burden (3.12), and ctDNA/MRD (3.08), which more frequently required explicit uncertainty statements and careful distinction between emerging, predictive, prognostic, and monitoring-related uses (Figure 4B). In particular, communication around PD-L1 and TMB should avoid presenting biomarker positivity as a guarantee of immunotherapy response, while FGFR alterations should be framed as molecular findings relevant to targeted therapy discussions rather than as direct immune-response markers.

Human review outcomes showed that none of the Qwen2-7B-Instruct outputs were classified as no edit. Of the 36 outputs, 21 outputs (58.3%) required minor edits, 13 outputs (36.1%) required major edits, and 2 outputs (5.6%) were classified as withhold/rewrite before any patient-facing use (Figure 4D). The most common revision issues were missing uncertainty statements (27.8%), lack of clinician referral (27.8%), confusion between diagnostic and predictive biomarkers (19.4%), overinterpretation of biomarker significance (13.9%), prognostic overstatement (11.1%), and unsupported treatment recommendation (5.6%).

These findings suggest that biomarker and precision pathology communication represents a higher-risk use case than general patient education or structured pathology explanation. A locally deployed LLM may help nurses translate complex molecular and immune-related terminology into patient-friendly language, but outputs involving PD-L1, TMB, ctDNA/MRD, or treatment implications require structured clinician oversight to prevent overinterpretation, unsupported recommendations, and insufficient communication of uncertainty.

### 3.5 Safety-critical performance in immune-related symptom scenarios

The symptom triage vignette set included 54 structured scenarios designed to evaluate red-flag recognition, escalation appropriateness, safety-netting, and nurse-review requirements in bladder cancer immunotherapy care. The scenarios covered low-risk routine symptoms (*n* = 8), moderate-risk symptoms (*n* = 12), high-risk immune-related adverse event scenarios (*n* = 16), postoperative or post-treatment complications (*n* = 10), and ambiguous scenarios requiring clinical-context interpretation (*n* = 8). Reference triage endpoints were defined using a four-level nursing-oriented framework: self-care or routine monitoring, contact with the nurse team within 24–48 h, urgent clinic or hospital assessment, and immediate emergency escalation.

**Figure 5:**
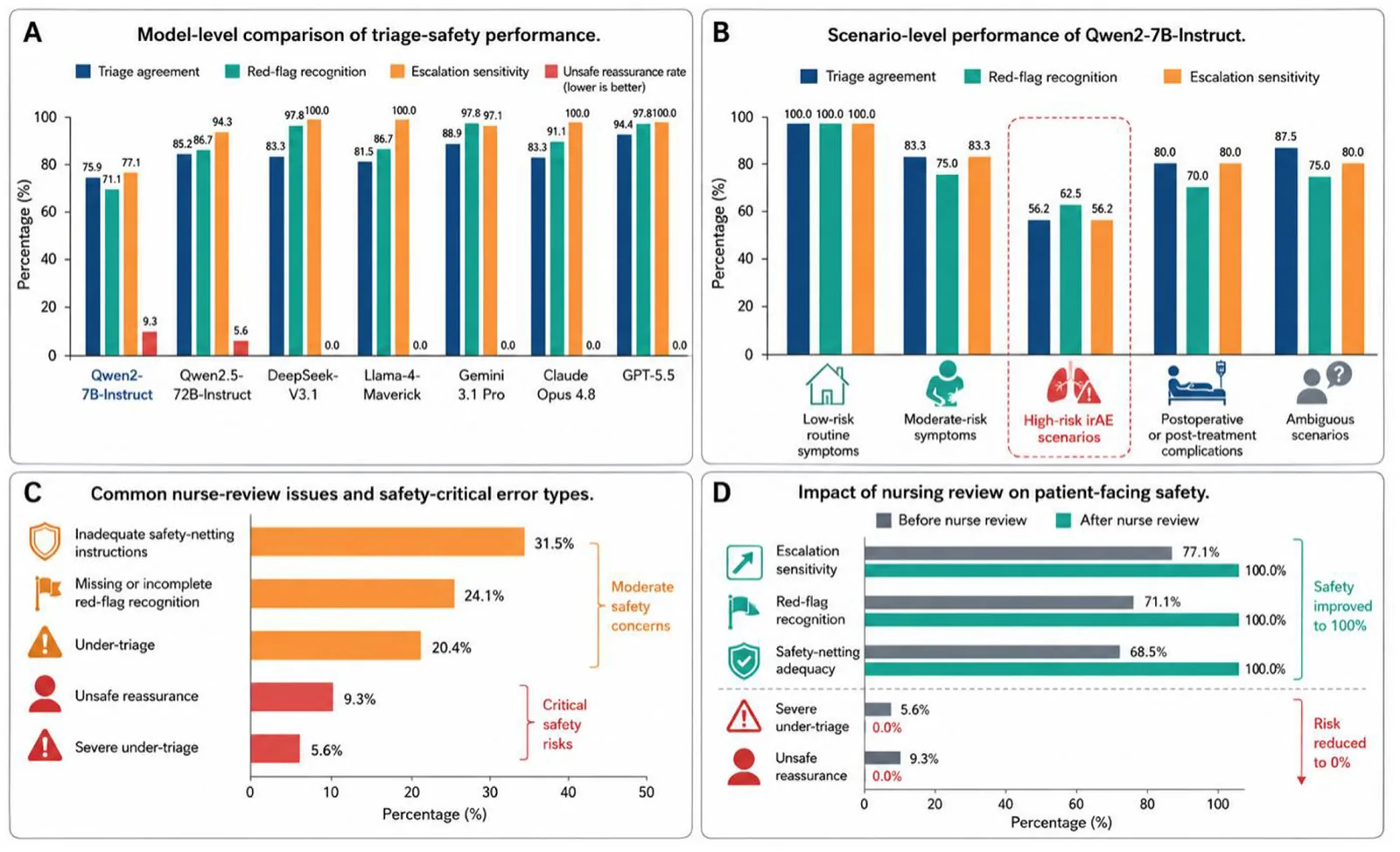
Safety-critical performance in immune-related symptom triage scenarios. **(A)** Model-level comparison of triage agreement, red-flag recognition, escalation sensitivity, and unsafe reassurance rate across the locally deployed Qwen2-7B-Instruct model and six comparator LLMs. **(B)** Scenario-level performance of Qwen2-7B-Instruct across low-risk routine symptoms, moderate-risk symptoms, high-risk immune-related adverse event scenarios, postoperative or post-treatment complications, and ambiguous scenarios. **(C)** Common nurse-review issues and safety-critical error types observed in Qwen2-7B-Instruct outputs. **(D)** Nursing review impact on escalation sensitivity, red-flag recognition, severe under-triage, unsafe reassurance, and safety-netting adequacy before and after human review/release control.

Across all triage scenarios, the locally deployed Qwen2-7B-Instruct model achieved a mean overall triage-safety score of 4.27 ± 0.65 on a 5-point scale. Its triage agreement with the expert reference standard was 75.9%, red-flag recognition was 71.1%, escalation sensitivity was 77.1%, and safety-netting adequacy was 68.5%. Under-triage occurred in 20.4% of outputs, including severe under-triage in 5.6%, and unsafe reassurance was observed in 9.3% of outputs. Comparator models showed higher safety performance, with triage agreement ranging from 81.5 to 94.4% and lower rates of unsafe reassurance and severe under-triage (Figure 5A; Table 5). Importantly, the overall triage-safety score and aggregate triage agreement should not be interpreted in isolation as indicators of safety in high-risk scenarios. Because the clinical consequences of under-triage are not equivalent across triage categories, performance in the high-risk irAE subset was considered separately.

**Table 5:** Performance of LLMs on symptom triage and safety-critical scenarios.

Model	Overall triage-safety score, mean ±SD	Triage agreement (%)	Red-flag recognition (%)	Escalation sensitivity (%)	Under-triage rate (%)	Severe under-triage rate (%)	Unsafe reassurance rate (%)	Safety-netting adequacy (%)	Major edit/rewrite (%)
Qwen2-7B-Instruct	4.27 ± 0.65	75.9	71.1	77.1	20.4	5.6	9.3	68.5	31.5
Qwen2.5-72B-Instruct	4.56 ± 0.32	85.2	86.7	94.3	7.4	0	5.6	87	20.4
DeepSeek-V3.1	4.59 ± 0.33	83.3	97.8	100	9.3	1.9	0	90.7	11.1
Llama-4-Maverick	4.58 ± 0.29	81.5	86.7	100	7.4	0	0	85.2	18.5
Gemini 3.1 Pro	4.69 ± 0.24	88.9	97.8	97.1	5.6	0	0	96.3	7.4
Claude Opus 4.8	4.63 ± 0.25	83.3	91.1	100	7.4	0	0	96.3	14.8
GPT-5.5	4.69 ± 0.20	94.4	97.8	100	1.9	0	0	98.1	3.7

Scenario-level analysis showed that Qwen2-7B-Instruct performed adequately in low-risk routine symptoms and postoperative or post-treatment complication scenarios, but performance decreased in high-risk immune-related adverse event scenarios. In the high-risk irAE subset, triage agreement was 56.2%, red-flag recognition was 62.5%, escalation sensitivity was 56.2%, under-triage was 43.8%, and unsafe reassurance was observed in 31.2% of outputs. The ambiguous scenario subset also required nursing oversight, with triage agreement of 87.5%, red-flag recognition of 75.0%, and escalation sensitivity of 80.0% (Figure 5B).

The most frequent nurse-review issues for Qwen2-7B-Instruct were inadequate safety-netting instructions (31.5%), missing or incomplete red-flag recognition (24.1%), under-triage (20.4%), unsafe reassurance (9.3%), and severe under-triage (5.6%). These errors were concentrated in scenarios involving dyspnea, severe diarrhea, jaundice, high fever with chills, altered mental status, heavy bleeding, and symptoms that were mild in intensity but persistent or occurring in a high-risk clinical context (Figure 5C).

Nurse review substantially changed the safety profile of Qwen2-7B-Instruct outputs. In the workflow, nurses added or clarified escalation instructions in outputs with missing red flags, insufficient safety-netting, or underestimated triage levels. After nurse review and release control, escalation sensitivity, red-flag recognition, and safety-netting adequacy were set to 100% for patient-facing outputs, while severe under-triage and unsafe reassurance were reduced to 0% through correction or withholding of unsafe responses (Figure 5D). Among Qwen2-7B-Instruct outputs, 3 of 54 (5.6%) required no edit, 34 of 54 (63.0%) required minor edits, 14 of 54 (25.9%) required major edits, and 3 of 54 (5.6%) were classified as withhold/rewrite.

These findings indicate that symptom triage is a safety-critical use case. Although the model showed moderate overall agreement with expert triage levels, this aggregate performance did not offset the lower agreement, reduced escalation sensitivity, under-triage, and unsafe reassurance observed in high-risk irAE scenarios. The locally deployed model may assist in generating preliminary nurse-reviewable drafts, but high-risk immune-related symptoms and ambiguous cases required structured nurse review, explicit escalation protocols, and clinician backup before any patient-facing release.

### 3.6 Pathology-risk-informed triage performance

The pathology-risk-informed triage set included 40 structured scenarios designed to determine whether LLMs could incorporate pathology-derived risk context into symptom triage and escalation guidance. Scenarios were grouped into five clinically relevant categories: high-grade T1 disease after intravesical BCG, muscle-invasive bladder cancer after surgery, lymph node-positive disease receiving systemic immune checkpoint inhibitor therapy, recurrent carcinoma *in situ*, and lymphovascular invasion or other high-risk pathology. Each scenario required the model to generate a nurse-reviewable triage response using the four-level framework applied in the symptom triage set.

**Figure 6:**
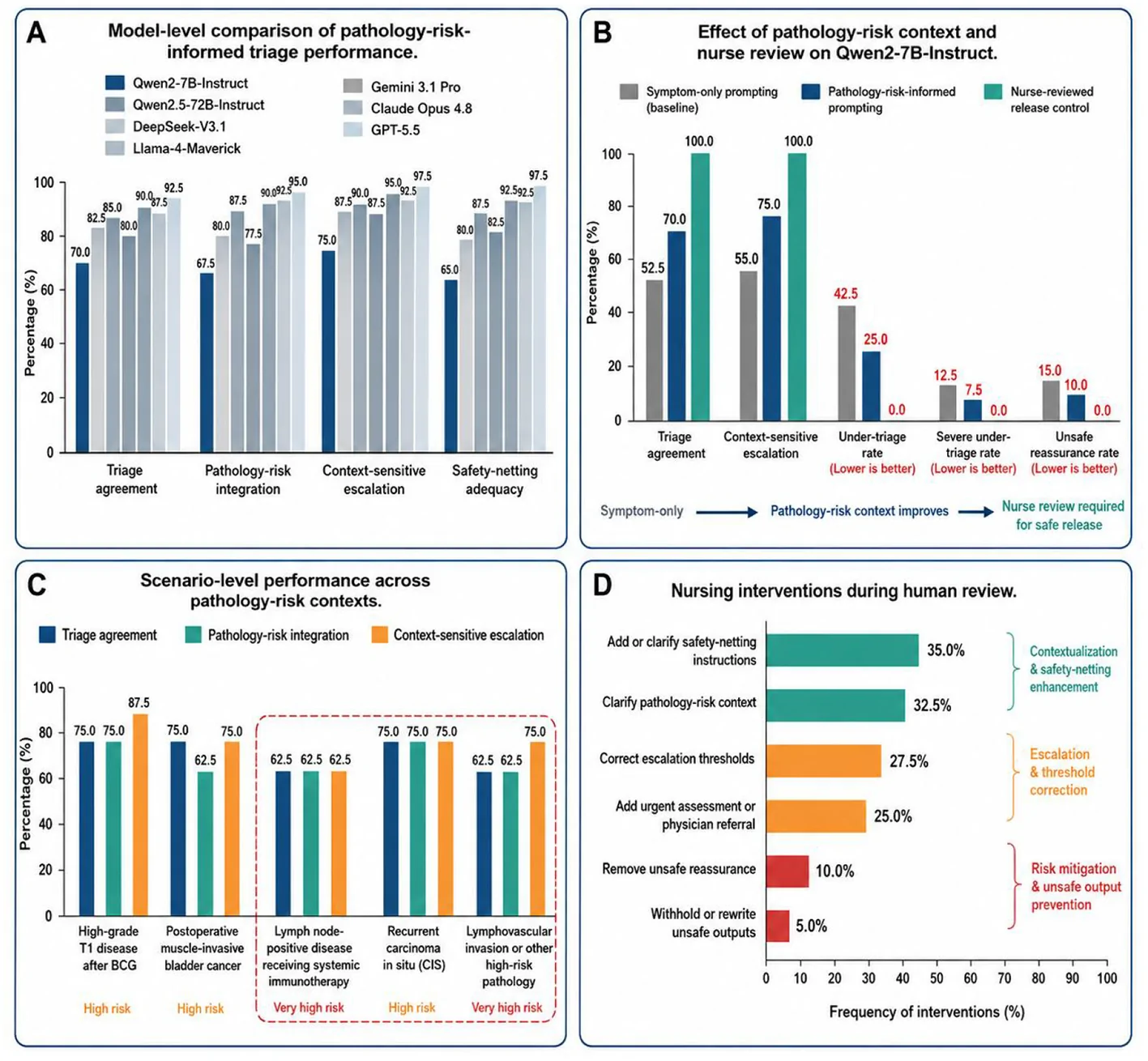
Pathology-risk-informed triage performance. **(A)** Model-level comparison of triage agreement, pathology-risk integration, context-sensitive escalation, and safety-netting adequacy across the locally deployed Qwen2-7B-Instruct model and six comparator LLMs. **(B)** Paired comparison of Qwen2-7B-Instruct outputs under symptom-only prompting, pathology-risk-informed prompting, and nurse-reviewed release control. **(C)** Scenario-level performance of Qwen2-7B-Instruct across five pathology-risk contexts: high-grade T1 disease after BCG, postoperative muscle-invasive bladder cancer, lymph node-positive disease receiving systemic immunotherapy, recurrent carcinoma *in situ*, and lymphovascular invasion or other high-risk pathology. **(D)** Nursing interventions applied during human review, including safety-netting clarification, pathology-risk contextualization, escalation-threshold correction, urgent referral addition, removal of unsafe reassurance, and withholding or rewriting unsafe outputs.

Across the 40 pathology-risk-informed triage scenarios, the locally deployed Qwen2-7B-Instruct model achieved a mean overall context-sensitive triage score of 4.45 ± 0.64 on a 5-point scale. Triage agreement with the expert reference standard was 70.0%, pathology-risk integration was 67.5%, and context-sensitive escalation was 75.0%. Under-triage occurred in 25.0% of outputs, including severe under-triage in 7.5%, while unsafe reassurance was observed in 10.0% of outputs. Safety-netting was adequate in 65.0% of outputs. Comparator models demonstrated higher overall performance, with triage agreement ranging from 80.0 to 92.5% and pathology-risk integration ranging from 77.5 to 95.0% (Figure 6A; Table 6).

**Table 6:** Performance of LLMs on pathology-risk-informed triage scenarios.

Model	Overall context score, mean ±SD	Triage agreement (%)	Pathology-risk integration (%)	Context-sensitive escalation (%)	Under-triage rate (%)	Severe under-triage rate (%)	Unsafe reassurance rate (%)	Safety-netting adequacy (%)	Major edit/rewrite (%)
Qwen2-7B-Instruct	4.45 ± 0.64	70	67.5	75	25	7.5	10	65	35
Qwen2.5-72B-Instruct	4.70 ± 0.46	82.5	80	87.5	12.5	2.5	5	80	20
DeepSeek-V3.1	4.80 ± 0.32	85	87.5	90	10	0	2.5	87.5	10
Llama-4-Maverick	4.71 ± 0.46	80	77.5	87.5	12.5	2.5	2.5	82.5	17.5
Gemini 3.1 Pro	4.87 ± 0.23	90	90	95	7.5	0	0	92.5	7.5
Claude Opus 4.8	4.87 ± 0.21	87.5	92.5	92.5	7.5	0	0	92.5	7.5
GPT-5.5	4.91 ± 0.17	92.5	95	97.5	5	0	0	97.5	2.5

A paired prompt comparison for Qwen2-7B-Instruct showed that adding pathology-risk context improved model behavior compared with symptom-only prompting. Triage agreement increased from 52.5% in symptom-only prompts to 70.0% in pathology-risk-informed prompts, and context-sensitive escalation increased from 55.0 to 75.0%. However, pathology-risk context did not eliminate safety problems: under-triage decreased from 42.5 to 25.0%, severe under-triage from 12.5 to 7.5%, and unsafe reassurance from 15.0 to 10.0%, indicating that pathology context improved but did not replace nursing oversight (Figure 6B).

Scenario-level analysis showed that Qwen2-7B-Instruct performed better in high-grade T1 after BCG and postoperative MIBC scenarios, with triage agreement of 75.0% in both categories. Performance was lower in lymph node-positive patients receiving systemic immunotherapy and in high-risk pathology with systemic symptoms, where triage agreement was 62.5% in each category. These lower-performing scenarios more often required recognition of immune-related pulmonary toxicity, systemic deterioration, infection risk, or the need to interpret mild symptoms in a high-risk pathology background. Recurrent CIS with worsening hematuria also required careful nursing review to prevent inappropriate reassurance and to ensure escalation advice was aligned with recurrence risk (Figure 6C).

Nursing review played a central safety-governance role. The most frequent nurse interventions were adding or clarifying safety-netting instructions (35.0%), clarifying pathology-risk context (32.5%), correcting escalation thresholds (27.5%), adding urgent assessment or physician referral instructions (25.0%), removing unsafe reassurance (10.0%), and withholding or rewriting unsafe outputs (5.0%). After nurse review and release control, patient-facing outputs were corrected to ensure complete escalation instructions, red-flag guidance, and appropriate clinical referral (Figure 6D).

These findings suggest that pathology-risk-informed triage is a clinically meaningful but safety-sensitive use case. The locally deployed LLM was able to incorporate some pathology-derived context into symptom triage, but it remained vulnerable to under-triage and insufficient risk contextualization in high-risk immunotherapy and postoperative scenarios. The model should therefore be used as a nurse-reviewed triage communication support tool rather than an autonomous escalation system.

### 3.7 Human review outcomes and revision burden

**Figure 7:**
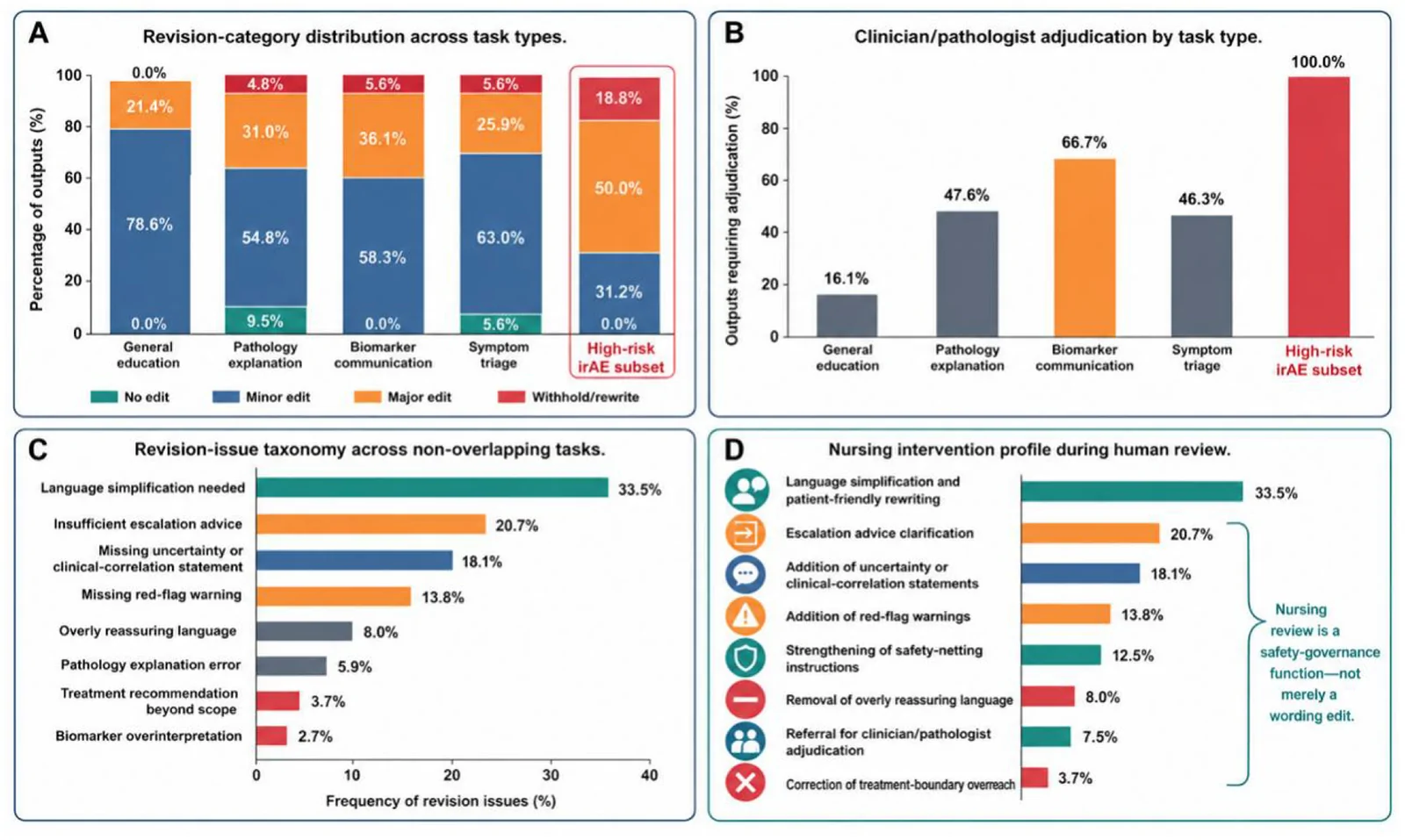
Human review outcomes and revision burden across task types. **(A)** Distribution of revision categories across general education, pathology explanation, biomarker communication, symptom triage, and the high-risk immune-related adverse event subset. **(B)** Proportion of outputs requiring clinician or pathologist adjudication by task type. **(C)** Overall taxonomy of revision issues identified during human review across non-overlapping Qwen2-7B-Instruct outputs. **(D)** Nursing intervention profile during human review, including language simplification, escalation advice clarification, red-flag warning addition, uncertainty or clinical-correlation statements, removal of unsafe reassurance, and referral or treatment-boundary correction.

Across the 188 non-overlapping Qwen2-7B-Instruct outputs evaluated in the patient education, pathology-informed communication, biomarker communication, and symptom triage tasks, only 7 outputs (3.7%) required no edit before potential patient-facing use. Most outputs required minor revision (122/188, 64.9%), whereas 52 outputs (27.7%) required major revision and 7 outputs (3.7%) were classified as withhold/rewrite (Figure 7A).

Initial revision classifications were concordant between the two nurse reviewers for 140 of 188 outputs (74.5%), whereas initial disagreement occurred for 48 outputs (25.5%). Clinician or pathologist adjudication was required for 78 outputs (41.5%). Importantly, adjudication frequency was higher than the initial disagreement rate because specialist adjudication was triggered not only by reviewer disagreement, but also by prespecified safety-critical, complex pathology, biomarker-related, and treatment-boundary concerns. As shown in Figure 7B and Table 7, Panel B, initial nurse agreement was highest for general education (89.3%) and lowest for biomarker communication (55.6%). Initial disagreement was most frequent in biomarker communication (44.4%), followed by pathology-informed communication (28.6%) and symptom triage (25.9%). Adjudication was most frequent for biomarker communication (66.7%), followed by pathology-informed communication (47.6%) and symptom triage (46.3%), and was least frequent for general education (16.1%).

**Table 7:** Human review outcomes and revision burden across task types.

Panel A. Revision burden and adjudication outcomes							
Task type	*n*	Clinician/ pathologist adjudication, *n* (%)	No edit, *n* (%)	Minor edit, *n* (%)	Major edit, *n* (%)	Withhold/ rewrite, *n* (%)	Major edit + withhold/rewrite, *n* (%)
Overall non-overlapping tasks	188	78 (41.5)	7 (3.7)	122 (64.9)	52 (27.7)	7 (3.7)	59 (31.4)
Patient education	56	9 (16.1)	0 (0.0)	44 (78.6)	12 (21.4)	0 (0.0)	12 (21.4)
Pathology-informed communication	42	20 (47.6)	4 (9.5)	23 (54.8)	13 (31.0)	2 (4.8)	15 (35.7)
Biomarker communication	36	24 (66.7)	0 (0.0)	21 (58.3)	13 (36.1)	2 (5.6)	15 (41.7)
Symptom triage	54	25 (46.3)	3 (5.6)	34 (63.0)	14 (25.9)	3 (5.6)	17 (31.5)
High-risk irAE subset^*^	16	16 (100.0)	0 (0.0)	5 (31.2)	8 (50.0)	3 (18.8)	11 (68.8)
**Panel B. Initial nurse-review consistency and adjudication patterns**							
**Task type**	***n***	**Initial nurse agreement**, ***n*** **(%)**	**Initial nurse disagreement**, ***n*** **(%)**	**Clinician/ pathologist adjudication**, ***n*** **(%)**	**Primary adjudication trigger(s)**		
Patient education	56	50 (89.3)	6 (10.7)	9 (16.1)	Reviewer disagreement and/or safety-critical warning-sign content		
Pathology-informed communication	42	30 (71.4)	12 (28.6)	20 (47.6)	Reviewer disagreement and/or complex pathology or treatment-boundary concerns		
Biomarker communication	36	20 (55.6)	16 (44.4)	24 (66.7)	Reviewer disagreement and/or uncertainty, overinterpretation, or treatment-boundary concerns		
Symptom triage	54	40 (74.1)	14 (25.9)	25 (46.3)	Reviewer disagreement and/or high-risk escalation concerns		
Overall non-overlapping tasks	188	140 (74.5)	48 (25.5)	78 (41.5)	—		
High-risk irAE subset^*^	16	9 (56.3)	7 (43.8)	16 (100.0)	Prespecified safety-critical adjudication regardless of initial agreement		

^*^The high-risk irAE subset (*n* = 16) is included within the symptom-triage group and was subject to prespecified clinician adjudication regardless of initial nurse-review agreement. irAE, immune-related adverse event.

Revision burden varied substantially by task type. General education had the lowest proportion of outputs requiring major revision or withhold/rewrite, with no outputs requiring withhold/rewrite and 12 of 56 outputs (21.4%) requiring major revision. Pathology-informed communication required greater oversight, with 13 of 42 outputs (31.0%) requiring major revision and 2 outputs (4.8%) requiring withhold/rewrite. Among the four non-overlapping task categories, biomarker communication showed the highest proportion of outputs requiring major revision or withhold/rewrite, with 15 of 36 outputs (41.7%) requiring these higher-intensity revisions. Symptom triage also required substantial review, with 17 of 54 outputs (31.5%) requiring major revision or withhold/rewrite. In the high-risk irAE subset, analyzed separately as a safety-critical subgroup nested within symptom triage, 11 of 16 outputs (68.8%) required major revision or withhold/rewrite. All high-risk irAE outputs underwent clinician adjudication according to the prespecified safety-review protocol.

The most frequent revision issues across the four non-overlapping task categories were language simplification needs (63/188, 33.5%), insufficient escalation advice (39/188, 20.7%), missing uncertainty or clinical-correlation statements (34/188, 18.1%), missing red-flag warnings (26/188, 13.8%), overly reassuring language (15/188, 8.0%), pathology explanation errors (11/188, 5.9%), treatment recommendations beyond the intended communication-support scope (7/188, 3.7%), and biomarker overinterpretation (5/188, 2.7%) (Figure 7C). These categories were not mutually exclusive and could co-occur within a single output.

Nursing review functioned as an active safety-governance layer rather than a superficial editing step. In addition to simplifying patient-facing language, nurses clarified escalation advice, added uncertainty or clinical-correlation statements, inserted red-flag warnings, strengthened safety-netting instructions, removed overly reassuring wording, and referred outputs with complex pathology, biomarker, treatment-boundary, or safety concerns for specialist review when required (Figure 7D). Overall, these findings indicate that the local model was relatively more suitable for generating nurse-reviewed drafts for routine, standardized educational content. However, this relative strength did not extend to warning-sign communication, biomarker communication, pathology-risk interpretation, or high-risk immune-related symptom triage, all of which required structured human-in-the-loop review and specialist backup before any potential patient-facing release.

## 4 Discussion

In this human-in-the-loop evaluation, the locally deployed Qwen2-7B-Instruct model showed task-dependent utility for bladder cancer immunotherapy communication, performing best in standardized education and structured pathology explanation but less reliably in biomarker interpretation, immune-related symptom escalation, and pathology-risk-informed triage. The central finding is that local deployment may improve data-governance feasibility, but clinical safety depended primarily on structured nurse review and clinician backup rather than on the model itself. Accordingly, the findings should be interpreted as evidence from a single-center, supervised communication-support workflow, rather than as validation of a new general evaluation methodology or of autonomous clinical communication.

The relatively stronger performance in general education is consistent with the known strengths of LLMs in generating readable and empathetic patient-facing explanations (15, 20). In our study, the model produced understandable responses for disease understanding, immunotherapy purpose, and home self-management, suggesting that LLMs may help reduce repetitive educational workload when outputs are reviewed before release. However, performance declined in warning-sign education, where the model more often required clarification of red flags and escalation thresholds. This distinction is clinically important because bladder cancer immunotherapy care includes both routine education and time-sensitive toxicity recognition. ASCO and ESMO guidelines emphasize early identification, grading, treatment interruption when appropriate, and urgent management of immune-related adverse events (6, 7). Therefore, high linguistic fluency should not be equated with escalation safety.

The pathology-informed communication results further show that the model was better at translating structured terms than at communicating risk and uncertainty. This pattern is plausible because pathology elements such as tumor grade, stage, muscle invasion, and lymph node status are relatively standardized, whereas carcinoma *in situ*, lymphovascular invasion, margin status, and treatment-context integration require judgment about prognosis, recurrence risk, and follow-up intensity (2, 21). The findings align with the current direction of uro-oncology, in which pathology-derived and molecular features increasingly inform treatment discussion and surveillance planning (22, 23). Our study adds a communication-focused perspective: the challenge is not only whether pathology information can guide care, but whether it can be explained to patients without overstatement or unauthorized treatment advice.

Biomarker communication was the most safety-sensitive explanatory task. The model could simplify terms such as PD-L1, TMB, FGFR alteration, ctDNA/MRD, and tumor immune microenvironment, but it frequently needed correction for uncertainty communication, clinician referral, and treatment-boundary awareness. This is consistent with the evolving status of biomarkers in urothelial carcinoma: molecular and immune markers may support stratification or therapeutic discussion, but many do not deterministically predict response for an individual patient (11). The model's tendency toward simplified explanations may benefit readability but risks compressing uncertainty into overly confident patient-facing statements. This supports the need for clinician-supervised biomarker communication, especially when patients may interpret biomarker positivity as a guarantee of response or prognosis.

The triage findings are perhaps the most clinically consequential. Although overall triage agreement was moderate, the model was less reliable in high-risk irAE scenarios, where under-triage and unsafe reassurance were observed. This is consistent with broader evidence that medical LLMs can perform well in constrained question-answering but remain vulnerable in dynamic, high-risk patient interaction tasks (15–17). The improvement observed after adding pathology-derived risk context indicates that structured contextual prompting can enhance model behavior. However, residual under-triage shows that prompt engineering alone is insufficient for safety-critical workflows. Nurse review corrected escalation thresholds, added red-flag warnings, strengthened safety-netting, and withheld unsafe outputs, indicating that nursing oversight functioned as an active safety-governance mechanism rather than a superficial editing step.

Previous oncology and urological studies have primarily evaluated LLM-generated responses to patient questions in terms of accuracy, readability, completeness, guideline concordance, or perceived usefulness (18, 24). In urological oncology, prior evaluations have examined patient-facing responses across prostate, bladder, kidney, and testicular cancers, as well as LLM-generated prostate cancer educational information assessed by clinicians and, in some studies, by patients (25, 26). These studies suggest that LLMs may assist with the generation of understandable cancer-related information, while also showing that response quality and clinical appropriateness vary across questions, models, and intended use settings (27).

Against this background, the present study should not be interpreted as introducing a new general evaluation framework. Rather, it is a single-center, application-specific evaluation of a locally deployed Qwen2-7B-Instruct model embedded in a predefined Mandarin-language, nurse-reviewed workflow for bladder cancer immunotherapy communication. The contribution of this study lies in delineating task-specific boundaries of use and the human oversight required within this setting: the model showed relatively greater utility for routine education and selected structured pathology explanations, whereas biomarker communication, pathology-risk interpretation, and immune-related symptom triage remained dependent on nurse review and clinician or pathologist backup. The revision-burden analysis was intended to characterize the practical level of review required before potential patient-facing use in this workflow, rather than to establish autonomous use, broad implementation readiness, or a generalisable evaluation method.

This study has several limitations. First, it was conducted within a single institution using a locally defined nurse-reviewed workflow; the observed review thresholds, adjudication practices, and communication priorities may not be directly transferable to other institutions. Second, the benchmarks were internally constructed structured Mandarin-language scenarios rather than prospectively collected real-world patient interactions, and therefore may not fully capture the linguistic variability, incomplete histories, emotional concerns, and evolving clinical context encountered in routine practice. Third, the study evaluated draft-generation performance and human revision burden rather than patient comprehension, clinician workload reduction, clinical outcomes, or the safety of direct patient-facing deployment. Finally, the findings relate primarily to the evaluated model, prompt structure, and institutional setting; external validation across additional centers, languages, models, and prospective clinical workflows is required before broader implementation can be considered.

Overall, these findings support a cautious implementation model in which locally deployed LLMs are used for draft generation in lower-risk educational tasks and structured pathology explanation, while biomarker communication and immune-related symptom triage remain under nurse-led review with clinician backup. Future studies should validate this workflow prospectively, test patient comprehension and workload outcomes, and develop institution-specific escalation protocols for safe LLM-assisted oncology nursing communication.

## Data Availability

The original contributions presented in the study are included in the article/Supplementary material, further inquiries can be directed to the corresponding authors.
