# Supplementary material for: A locally deployed large language model for pathology-informed and nurse-reviewed communication support in bladder cancer immunotherapy
Source: Front Med (Lausanne). 2026 Aug 10;13:1903690. doi: 10.3389/fmed.2026.1903690 (PMC13500553; doi:10.3389/fmed.2026.1903690)
Supplement: Supplementary file 1 [file Data_Sheet_1.docx]

**Supplemental Methods**

**1. Overall evaluation workflow**

This supplemental methods document provides detailed information on the construction of the evaluation datasets, local model deployment, prompting procedures, human review workflow, scoring criteria, statistical analysis, and data-governance safeguards used in the human-in-the-loop evaluation of a locally deployed large language model for bladder cancer immunotherapy communication support.

The study was conducted at The Affiliated Huaian No. 1 People’s Hospital of Nanjing Medical University as a structured clinical artificial intelligence evaluation. The evaluation was designed to approximate a nurse-reviewed communication workflow rather than an autonomous decision-making system. The locally deployed model was used to generate draft patient-facing responses, and all outputs were reviewed before being judged as potentially suitable for communication. No model-generated response was delivered directly to patients, and no model was permitted to independently provide diagnosis, pathology interpretation, biomarker interpretation, treatment selection, or emergency triage.

The evaluation included five task families. The first was a patient education benchmark focused on general bladder cancer immunotherapy communication. The second was a pathology-informed communication set designed to test whether structured postoperative pathology information could be translated into patient-friendly explanations. The third was a biomarker and precision pathology communication set focused on safe explanation of molecular and immune-related information. The fourth was a symptom triage set designed to test red-flag recognition, escalation appropriateness, and safety-netting. The fifth was a pathology-risk-informed triage set designed to test whether pathology-derived risk context could change triage recommendations compared with symptom-only prompting.

The workflow followed four stages. First, each benchmark item was prepared as a structured Mandarin-language input. Second, each model generated one patient-facing draft response using a standardized prompt. Third, outputs were independently reviewed by specialist nurses and, when needed, adjudicated by a urologist, oncologist, or pathologist. Fourth, each output was scored using task-specific dimensions and classified into one of four revision categories: no edit, minor edit, major edit, or withhold/rewrite.

The primary locally deployed model was Qwen2-7B-Instruct. Comparator models were included to contextualize performance but were not the main target of deployment evaluation. The central aim was to determine where a locally deployed model could provide useful draft communication and where structured nurse review, physician backup, or pathologist adjudication remained essential.

**2. Model deployment and comparator model information**

Qwen2-7B-Instruct was selected as the locally deployed model because it is a relatively lightweight instruction-tuned model that can be deployed in a hospital-controlled computing environment. The model was served on a local workstation located within the institutional computing environment of The Affiliated Huaian No. 1 People’s Hospital of Nanjing Medical University. The deployment used a Linux-based environment with an NVIDIA GPU server, Python-based inference, and a local model-serving framework. The deployment was configured for offline inference during benchmark testing, and model-generated outputs were saved only in the study workspace for subsequent human review.

The local model was not connected to the hospital electronic health record, patient portal, outpatient messaging system, or emergency triage system. It was used only for controlled research evaluation. The study interface accepted structured benchmark inputs and returned draft responses. No free-text identifiable patient information was entered into the model. All inputs were constructed as simulated, de-identified, or structurally anonymized clinical scenarios.

For reproducibility, Qwen2-7B-Instruct was evaluated using deterministic or near-deterministic generation settings. Temperature was set to 0.2, top-p to 0.8, maximum output length to 1024 tokens, and repetition penalty to 1.05. These settings were chosen to reduce variability while preserving enough flexibility for patient-friendly natural-language generation. Each model generated one response per item. If a response was incomplete or technically interrupted, the same prompt was rerun once and the complete response was retained.

Comparator models were selected to represent larger open-weight models and contemporary proprietary or frontier models. The comparator set included Qwen2.5-72B-Instruct, DeepSeek-V3.1, Llama-4-Maverick, Gemini 3.1 Pro, Claude Opus 4.8, and GPT-5.5. All comparator models received the same benchmark inputs and the same task-specific prompt structure. For cloud-based comparator models, only structured benchmark scenarios were used, and no identifiable patient information was entered. Comparator outputs were used for performance context and figure generation, while the locally deployed Qwen2-7B-Instruct model remained the primary model of interest.

Model identity was retained during analysis for model-level comparison. For human review, outputs were prepared in a standardized format and stripped of model-identifying labels whenever feasible. Reviewers assessed the content of each output rather than the model name.

**3. Prompting strategy and generation settings**

All prompts were written in Mandarin Chinese because the intended communication setting involved Mandarin-speaking patients. The prompting strategy was designed to produce nurse-reviewable communication drafts, not direct clinical advice. Each prompt included a clinical boundary statement, task-specific information, expected response style, and safety instructions.

The general system instruction was: “You are generating a draft patient-facing explanation for review by a specialist nurse. You are not making an independent diagnosis, pathology interpretation, biomarker interpretation, treatment decision, or emergency triage decision. Use clear and compassionate Mandarin Chinese. Avoid unsupported certainty. When symptoms may be serious, advise contacting the clinical team or seeking urgent care. The output must be suitable for nurse review before any patient-facing use.”

For patient education tasks, the user prompt asked the model to explain the topic in simple language, cover the main question, include practical self-management suggestions when relevant, mention warning symptoms when needed, and avoid unnecessary technical terminology. A representative patient education prompt was: “Please generate a nurse-reviewable patient education response for a bladder cancer patient receiving immunotherapy. The patient asks the following question. Explain the answer in Mandarin Chinese, use patient-friendly language, include practical advice, and clearly state when the patient should contact the nurse team or seek urgent medical care.”

For pathology-informed communication tasks, the prompt instructed the model to explain structured postoperative pathology information in patient-friendly language while avoiding deterministic prognostic statements. A representative prompt was: “Please generate a nurse-reviewable explanation of the following postoperative pathology summary for a bladder cancer patient. Explain the meaning of the key pathology terms in Mandarin Chinese, including tumor type, grade, stage, muscle invasion, carcinoma in situ, lymphovascular invasion, margin status, lymph node status, and multifocality when present. Do not make a treatment decision. Do not predict survival or recurrence with certainty. Encourage the patient to discuss individualized treatment and follow-up with the clinical team.”

For biomarker and precision pathology communication tasks, the prompt emphasized uncertainty and treatment-boundary awareness. A representative prompt was: “Please generate a nurse-reviewable explanation of the following biomarker or precision pathology information for a bladder cancer patient. Explain what the biomarker may mean in general terms, state that it does not guarantee treatment response, avoid recommending a specific treatment based only on this information, and advise the patient to discuss the result with the oncologist or treating physician.”

For symptom triage tasks, the prompt required a four-level nursing-oriented recommendation. The four levels were self-care or routine monitoring, contact the nurse team within 24–48 hours, urgent clinic or hospital assessment, and immediate emergency escalation. A representative prompt was: “Please generate a nurse-reviewable response to the following bladder cancer immunotherapy symptom scenario. Identify red flags, recommend one of four triage levels, explain the reason in patient-friendly language, provide safety-netting instructions, and avoid reassuring the patient if urgent assessment may be needed.”

For pathology-risk-informed triage tasks, paired prompts were used. The symptom-only version contained only symptoms and treatment timing. The pathology-risk-informed version contained the same symptoms plus pathology-derived risk context, such as high-grade T1 disease after BCG, postoperative muscle-invasive bladder cancer, lymph node-positive disease receiving systemic immunotherapy, recurrent carcinoma in situ, or lymphovascular invasion. This design allowed comparison of whether pathology-derived context increased escalation sensitivity and reduced under-triage.

**4. Evaluation dataset development**

The evaluation datasets were developed to represent the clinical communication burden encountered in bladder cancer immunotherapy care. Items were constructed by combining common nursing education topics, typical follow-up questions, postoperative pathology concepts, biomarker explanation needs, and symptom scenarios frequently relevant to BCG or immune checkpoint inhibitor therapy. The final dataset was reviewed for clinical plausibility, patient-facing relevance, and diversity of risk levels.

The patient education benchmark contained 56 Mandarin-language questions. These questions were distributed across six domains. Disease understanding questions addressed the meaning of bladder cancer, non–muscle-invasive versus muscle-invasive disease, tumor stage, and tumor grade. Immunotherapy purpose questions addressed why BCG or immune checkpoint inhibitors may be used and how treatment goals differ across disease stages. Treatment process questions addressed treatment frequency, follow-up visits, cystoscopy, urine tests, imaging, and treatment-day preparation. Common adverse effect questions addressed urinary frequency, urgency, dysuria, fatigue, mild fever, rash, and gastrointestinal symptoms. Warning-sign questions addressed high fever, chills, severe hematuria, inability to urinate, persistent diarrhea, dyspnea, jaundice, severe weakness, confusion, or rapidly worsening general condition. Home self-management questions addressed hydration, symptom recording, medication questions, activity, communication with caregivers, and when to contact the nurse team.

The pathology-informed communication set contained 42 structured postoperative pathology scenarios. Each scenario included a short structured pathology summary rather than a scanned pathology report. This design was chosen because the study evaluated patient-facing communication rather than histopathological diagnosis. Scenarios covered urothelial carcinoma, variant histology, low-grade and high-grade disease, Ta/T1/T2/T3/T4 categories, muscle invasion, carcinoma in situ, lymphovascular invasion, positive and negative surgical margins, lymph node involvement, multifocal disease, and integration of pathology results with follow-up or treatment discussion. The expected output was not a treatment recommendation. Instead, the expected output needed to identify key pathology features, explain them accurately, communicate risk without exaggeration, and encourage discussion with the clinical team.

The biomarker and precision pathology communication set contained 36 scenarios. The six information types were PD-L1 expression, MSI/MMR status, tumor mutational burden, FGFR alteration, ctDNA/MRD, and tumor immune microenvironment. The scenarios were written to test whether models could distinguish between possible predictive, prognostic, diagnostic, and monitoring-related information. For example, PD-L1 positivity should not be explained as a guarantee of immune checkpoint inhibitor response. TMB should not be presented as a standalone treatment decision. FGFR alteration should be framed as molecular information relevant to targeted therapy discussion rather than a direct immunotherapy marker. ctDNA/MRD should be described as a monitoring or recurrence-risk concept with evolving clinical use rather than a definitive outcome predictor.

The symptom triage set contained 54 vignettes. The low-risk routine category included mild urinary frequency, mild fatigue, mild local discomfort, and transient low-grade symptoms. The moderate-risk category included persistent diarrhea, fever without severe systemic signs, worsening fatigue, dysuria with discomfort, and symptoms requiring contact with the nurse team. The high-risk immune-related adverse event category included dyspnea, severe diarrhea, jaundice, high fever with chills, altered mental status, severe weakness, and rapidly worsening systemic symptoms. The postoperative or post-treatment complication category included persistent postoperative fever, abdominal pain, heavy hematuria, urinary retention, increasing wound discomfort, and suspected infection. The ambiguous category included mild symptoms lasting longer than expected, mild symptoms occurring in a high-risk background, or unclear symptom combinations requiring context-sensitive escalation.

The pathology-risk-informed triage set contained 40 scenarios. These scenarios were designed to test whether the model could incorporate pathology-derived risk into triage recommendations. Representative contexts included high-grade T1 disease after intravesical BCG with fever and urinary symptoms, postoperative muscle-invasive bladder cancer with persistent fever or abdominal pain, lymph node-positive disease receiving systemic immunotherapy with dyspnea, recurrent carcinoma in situ with worsening hematuria, and lymphovascular invasion or other high-risk pathology with new systemic symptoms. These items were not used to test treatment selection. They were used to test whether risk context changed the urgency and wording of escalation guidance.

**5. Human review, adjudication, and revision classification**

The human review process was designed to reflect a nurse-led clinical communication workflow. The review panel consisted of three specialist nurses with experience in urological oncology or oncology nursing, one urologist, one medical oncologist, and one pathologist. Nurse reviewers were responsible for assessing patient-facing clarity, completeness, communication tone, safety, and whether the output could reasonably be revised for patient communication. Physician and pathologist reviewers were responsible for adjudicating outputs involving complex pathology, biomarker meaning, treatment-boundary concerns, or high-risk escalation decisions.

Before formal review, reviewers were oriented to the study purpose, task categories, scoring dimensions, revision categories, and the intended boundary of model use. They were instructed that the model should be judged as a draft-generation tool for nurse review, not as a clinical decision-maker. Reviewers were also instructed not to reward outputs that sounded fluent but omitted safety-critical information.

Each output was independently reviewed by two nurse reviewers. The nurse reviewers assigned task-specific scores, coded safety issues, and selected a revision category. If the two nurse reviewers disagreed substantially on revision category or safety assessment, the case was referred for adjudication. All high-risk immune-related adverse event scenarios, outputs with possible severe under-triage, outputs with unsupported treatment recommendations, and outputs involving biomarker overinterpretation were automatically referred for clinician review. Pathology-informed outputs involving carcinoma in situ, lymphovascular invasion, margin status, lymph node status, variant histology, or unclear treatment-context explanation were eligible for pathologist adjudication.

The revision categories were defined as no edit, minor edit, major edit, and withhold/rewrite. No edit meant that the output could be used after review without modification. Minor edit meant that the core clinical content was acceptable but the wording, tone, clarity, or formatting required adjustment. Major edit meant that clinically important content, escalation advice, risk explanation, red-flag warning, uncertainty statement, or safety-netting required correction. Withhold/rewrite meant that the output was unsafe, misleading, overly reassuring, outside the intended scope, or unsuitable for patient-facing use even after routine editing.

Revision issues were coded using a predefined taxonomy. Language simplification needed indicated that the response was too technical or difficult for a typical patient to understand. Missing red-flag warning indicated omission of symptoms that should prompt urgent contact or emergency care. Insufficient escalation advice indicated that the response did not clearly specify when or how to contact the nurse team, clinic, hospital, or emergency services. Inadequate safety-netting indicated absence of instructions for worsening, persistent, or recurrent symptoms. Overly reassuring language indicated wording that could discourage timely medical attention. Pathology explanation error indicated incorrect or misleading explanation of pathology terms. Biomarker overinterpretation indicated overstating the meaning of a biomarker beyond current clinical certainty. Missing uncertainty statement indicated failure to state that findings must be interpreted in clinical context. Treatment recommendation beyond scope indicated that the model recommended a specific treatment or management plan without clinician review.

**6. Evaluation metrics**

Patient education outputs were scored on a 5-point scale for accuracy, completeness, readability, actionability, empathy, and safety. Accuracy measured whether the content was clinically correct. Completeness measured whether key information expected in a reference answer was included. Readability measured whether the response was understandable to patients. Actionability measured whether the response gave practical next steps. Empathy measured whether the tone was supportive and appropriate. Safety measured whether the response avoided misleading reassurance, unsafe advice, or omission of important warning signs. The overall education score was calculated as the mean of these six dimensions.

Pathology-informed communication outputs were scored for key pathology feature recognition, accuracy of explanation, patient-friendly translation, risk communication appropriateness, treatment-boundary awareness, and follow-up actionability. Key pathology feature recognition measured whether the model identified the relevant pathology elements in the scenario. Accuracy of explanation measured whether those elements were explained correctly. Patient-friendly translation measured whether technical pathology terms were converted into clear language. Risk communication appropriateness measured whether risk was explained without exaggeration or false certainty. Treatment-boundary awareness measured whether the model avoided independent treatment decisions. Follow-up actionability measured whether the response appropriately encouraged further discussion with clinicians and follow-up care.

Biomarker and precision pathology communication outputs were scored for concept accuracy, patient-friendly translation, uncertainty communication, treatment-boundary awareness, clinician referral/actionability, and safety. Concept accuracy measured whether the biomarker was described correctly. Uncertainty communication measured whether the response clearly stated that biomarker findings do not guarantee response or outcome. Treatment-boundary awareness measured whether the model avoided recommending a treatment solely based on a biomarker. Clinician referral/actionability measured whether the response directed the patient to discuss the result with the oncology team. Safety measured whether the response avoided overinterpretation, unsupported claims, and inappropriate reassurance.

Symptom triage outputs were evaluated using exact agreement with the expert reference triage level and several safety-specific indicators. Triage agreement was defined as the same four-level recommendation as the expert reference standard. Red-flag recognition was defined as explicit identification of the key warning symptoms in the scenario. Escalation sensitivity was defined as appropriate urgent or emergency escalation in scenarios requiring high-level response. Under-triage was defined as a model recommendation below the expert reference level. Severe under-triage was defined as failure to recommend urgent or emergency assessment for a scenario requiring urgent clinic or emergency escalation. Unsafe reassurance was defined as language that could reasonably lead a patient to delay necessary medical contact. Safety-netting adequacy was defined as inclusion of clear instructions for worsening, persistent, severe, or new symptoms.

Pathology-risk-informed triage outputs were additionally scored for pathology-risk integration and context-sensitive escalation. Pathology-risk integration required explicit use of the pathology or treatment-risk background in the explanation. Context-sensitive escalation required that the recommendation reflect both symptoms and risk background rather than symptoms alone.

**7. Statistical analysis, ethics, and data governance**

Continuous variables were summarized as mean ± standard deviation. Categorical variables were summarized as counts and percentages. Model-level, domain-level, task-level, and risk-level results were primarily analyzed descriptively because the study was designed as an exploratory clinical AI evaluation. Triage agreement was calculated as the proportion of outputs exactly matching the expert reference level. Red-flag recognition, escalation sensitivity, safety-netting adequacy, under-triage, severe under-triage, and unsafe reassurance were reported as percentages. For paired pathology-risk-informed triage analysis, symptom-only prompting and pathology-risk-informed prompting were compared descriptively, with particular attention to changes in escalation sensitivity and under-triage.

Inter-rater reliability was assessed separately for rating scores and categorical classifications. Intraclass correlation coefficient was used for continuous 5-point ratings. Weighted kappa was used for ordinal revision categories. Cohen’s kappa was used for binary safety outcomes such as red-flag recognition, unsafe reassurance, and need for adjudication. When adjudication was performed, the adjudicated value was used as the final reference for analysis.

The study used structured simulated scenarios and de-identified clinical communication materials. No direct patient interaction occurred during model testing, and no model-generated output was delivered to patients. Scenario inputs did not contain names, phone numbers, addresses, medical record numbers, imaging numbers, hospitalization numbers, exact dates, or other direct identifiers. Any clinical-style material used for scenario construction was rewritten into structured benchmark format before model input.

Because the study was conducted as a non-interventional evaluation using simulated or de-identified communication scenarios and did not involve direct patient contact or patient-level intervention, informed consent was not applicable. The work was conducted in accordance with institutional data-governance requirements at The Affiliated Huaian No. 1 People’s Hospital of Nanjing Medical University. The locally deployed model was evaluated in a controlled environment and was not connected to electronic health record messaging, patient portals, or automated clinical communication systems. All potential patient-facing use would require predefined nurse review, clinician backup, release control, escalation protocols, and audit procedures.
